# Regulation of SESAME-mediated H3T11 phosphorylation by glycolytic enzymes and metabolites

**DOI:** 10.1371/journal.pone.0175576

**Published:** 2017-04-20

**Authors:** Qi Yu, Chong Tong, Mingdan Luo, Xiangyan Xue, Qianyun Mei, Lixin Ma, Xiaolan Yu, Wuxiang Mao, Lingbao Kong, Xilan Yu, Shanshan Li

**Affiliations:** 1 Hubei Collaborative Innovation Center for Green Transformation of Bio-resources,College of Life Sciences, Hubei University, Wuhan, Hubei, China; 2 Department of HumanPopulation Genetics, Human Aging Research Institute and School of Life Science, Nanchang University, Nanchang, China; University of Nebraska Medical Center, UNITED STATES

## Abstract

Cancer cells prefer aerobic glycolysis, but little is known about the underlying mechanism. Recent studies showed that the rate-limiting glycolytic enzymes, pyruvate kinase M2 (PKM2) directly phosphorylates H3 at threonine 11 (H3T11) to regulate gene expression and cell proliferation, revealing its non-metabolic functions in connecting glycolysis and histone modifications. We have reported that the yeast homolog of PKM2, Pyk1 phosphorylates H3T11 to regulate gene expression and oxidative stress resistance. But how glycolysis regulates H3T11 phosphorylation remains unclear. Here, using a series of glycolytic enzyme mutants and commercial available metabolites, we investigated the role of glycolytic enzymes and metabolites on H3T11 phosphorylation. Mutation of glycolytic genes including phosphoglucose isomerase (*PGI1*), enolase (*ENO2*), triosephosphate isomerase (*TPI1*), or folate biosynthesis enzyme (*FOL3*) significantly reduced H3T11 phosphorylation. Further study demonstrated that glycolysis regulates H3T11 phosphorylation by fueling the substrate, phosphoenonylpyruvate and the coactivator, FBP to Pyk1. Thus, our results provide a comprehensive view of how glycolysis modulates H3T11 phosphorylation.

## Introduction

Glycolysis is the fundamental metabolism highly conserved in most organisms, which comprises a series of enzymatic steps that sequentially convert glucose to pyruvate. In the presence of oxygen, most pyruvate undergoes oxidative phosphorylation to generate ATP in mitochondria; while in the absence of oxygen, pyruvate is converted to lactate with few ATP produced [1,2]. However, cancer cells preferentially convert pyruvate to lactate even in the presence of oxygen, a phenomenon known as “Warburg effect” or aerobic glycolysis [1]. Aerobic glycolysis enables cells to accumulate a large amount of glycolytic intermediates, which serve as building blocks to meet cell rapid growth and division [1–4]. Nevertheless, it remains poorly understood about why tumor cells prefer accelerated glycolysis and reduced oxidative phosphorylation.

Emerging evidence showed that most glycolytic enzymes are deregulated in cancer cells and plays important roles in tumorigenesis [2,5]. All essential glycolytic enzymes can be translocated into nucleus where they participate in tumor progression independent of their canonical metabolic roles [6]. One such non-metabolic function is catalyzing and/or modulating histone modifications. The typical example is tumor specific pyruvate kinase M2 (PKM2), which plays important roles in cancer metabolism rewiring [7]. Yang et al. reported that in human glioblastoma multiforme cells, PKM2 translocates into nucleus upon epidermal growth factor (EGF) receptor activation, where it phosphorylates histone H3 at threonine 11 (H3T11), which is required for dissociation of histone deacetylase 3 (HDAC3) from the promoter regions of *CCDN1*(encoding cyclin D1) and *MYC*, leading to their activation, tumor cell proliferation, cell-cycle progression, and brain tumorigenesis [8]. Previously, we have reported that Pyk1, the yeast homologue of PKM2 also has some non-metabolic functions [9]. Similar to PKM2, Pyk1 can phosphorylate H3T11 in vivo and in vitro and this protein kinase activity is regulated by serine metabolic pathway [9]. Specifically, H3T11 phosphorylation is regulated by enzymes involved in serine metabolism including Ser1, Ser2, Ser33, Shm2, Met6 and Met13. Moreover, by combining protein purification technique with mass spectrometry, we found that Pyk1 forms a novel complex, SESAME (serine responsive SAM-containing metabolic enzyme complex) with other metabolic enzymes, including Sam1, Sam2, Ser33, Shm2 and Acs2 [9]. Further studies showed that SESAME interacts with Set1 complex, which methylates H3K4. By supplying the cofactor SAM for Set1 complex, SESAME regulates both H3K4me3 and H3T11 phosphorylation. As a consequence, SESAME regulates gene expression and cell resistance to oxidative stress [9].

Cellular metabolism regulates histone modifications and many metabolites serve as essential cofactors for chromatin-modifying enzymes to control the transcription or translation processes [2,10,11]. For example, about 5% glucose is used for hexosamine biosynthetic pathways to produce GlcNAc, which is the donor for histone glycosylation [12]. Through glycolysis, glucose can be converted to acetyl CoA, along with decreased NAD^+^/NADH, which in turn regulate the activity of histone acetyltransferases and histone deacetylases as well as the chromatin structure [10,12–14]. We have previously shown that glucose is required for SESAME to phosphorylate H3T11 [9]; however, the pathways and metabolites critical for H3T11 phosphorylation remain poorly defined. Here, we analyzed the function of glycolytic metabolic enzymes and metabolites on H3T11 phosphorylation.

## Materials and methods

### Cells and growth conditions

All yeast strains used in this study are described in Table 1. All yeast cells were grown in YPD (2% yeast extract, 1% peptone, 2% glucose) medium unless otherwise indicated. For doxycycline treatment, WT Tet and mutants were grown in YPD to an OD600 of 0.7 and then treated with 0, 12.5, 25 and 50 μg/ml of doxycycline.

**Table 1:** List of strains used in this study.

Name	Genotype	Source
BY4741	*MATa his3Δ1 leu2Δ0 met15Δ0 ura3Δ0*	
*sam1Δ*	*MATa his3Δ1 leu2Δ0 met15Δ0 ura3Δ0 sam1Δ*::*KAN*	Open Biosystems
*sam2Δ*	*MATa his3Δ1 leu2Δ0 met15Δ0 ura3Δ0 sam2Δ*::*KAN*	Open Biosystems
*ser33Δ*	*MATa his3Δ1 leu2Δ0 met15Δ0 ura3Δ0 ser33Δ*::*KAN*	Open Biosystems
*acs1Δ*	*MATa his3Δ1 leu2Δ0 met15Δ0 ura3Δ0 acs1Δ*::*KAN*	Open Biosystems
*shm2Δ*	*MATa his3Δ1 leu2Δ0 met15Δ0 ura3Δ0 shm2Δ*::*KAN*	Open Biosystems
*eno1Δ*	*MATa his3Δ1 leu2Δ0 met15Δ0 ura3Δ0 eno1Δ*::*KAN*	Open Biosystems
*pdc1Δ*	*MATa his3Δ1 leu2Δ0 met15Δ0 ura3Δ0 pdc1Δ*::*KAN*	Open Biosystems
WT Tet (R1158)	*MATa his3-1 leu2-0 met15-0 URA3*::*CMV-tTA*	Yeast Tet-promoters Hughes Collection
TetO_7_-*ENO2*	*MATa his3-1 leu2-0 met15-0 pENO2*::*kanR-tet07-TATA URA3*::*CMV-tTA*	Yeast Tet-promoters Hughes Collection
TetO_7_-*TPI1*	*MATa his3-1 leu2-0 met15-0 pTPI1*::*kanR-tet07-TATA URA3*::*CMV-tTA*	Yeast Tet-promoters Hughes Collection
TetO_7_-*PGI1*	*MATa his3-1 leu2-0 met15-0 pPGI1*::*kanR-tet07-TATA URA3*::*CMV-tTA*	Yeast Tet-promoters Hughes Collection
TetO_7_-*FOL3*	*MATa his3-1 leu2-0 met15-0 pFOL3*::*kanR-tet07-TATA URA3*::*CMV-tTA*	Yeast Tet-promoters Hughes Collection

### Histone extraction

Histones were extracted from yeast cells as described previously [9,15]. Briefly, cells grown in 5 ml culture was harvested and lysed in 2M NaOH with 8% β-mercaptoethanol. Cell lysate was centrifuged at 13,000 rpm for 2 min and the pellet was washed three times with TAP extraction buffer (40 mM HEPES-KOH pH7.5, 10% glycerol, 350 mM NaCl, 0.1% Tween-20). Cell pellets were resuspended in 1× SDS-sample buffer.

### Western blots analysis

Protein samples were separated by 15% SDS-PAGE and transferred to Immobilon-P PVDF membrane (Merck Millipore). The blots were probed with antibodies against H3 (Abclonal Biotechnology) and H3pT11 (Abcam, ab5168) followed by incubation with horseradish peroxidase-labeled IgG secondary antibodies (Abclonal Biotechnology). The specific proteins were visualized by using the ECL Chemiluminescence Detection Kit (Amersham Biosciences). Western blots were quantified with ImageJ software.

### Quantitative reverse-transcription PCR (qRT-PCR)

10 ml cultures were grown in YPD to an OD_600_ of 0.6–0.8 and treated with doxycycline for 0.5 hour. Total RNA was isolated and Real-Time RT-PCR was performed with SYBR green and gene specific primers as described previously [9]. All transcripts quantities were normalized against the amount of *ACT1* transcript. Primers used were listed in S1 Table.

### Analysis of fructose 1, 6-biphosphate (FBP)

50 ml cells were grown in YPD to an OD_600_ of ~1.0 and treated with 50 μg/μl doxycycline for 3 hours. Cells were harvested and lysed with glass beads. The intracellular FBP concentrations were determined using FBP analysis kit according to the protocol recommended by the manufacturer (Comin Biotechnology Co., Ltd, Suzhou).

## Results

### Glucose is required for SESAME-mediated H3T11 phosphorylation

**Fig 1:**
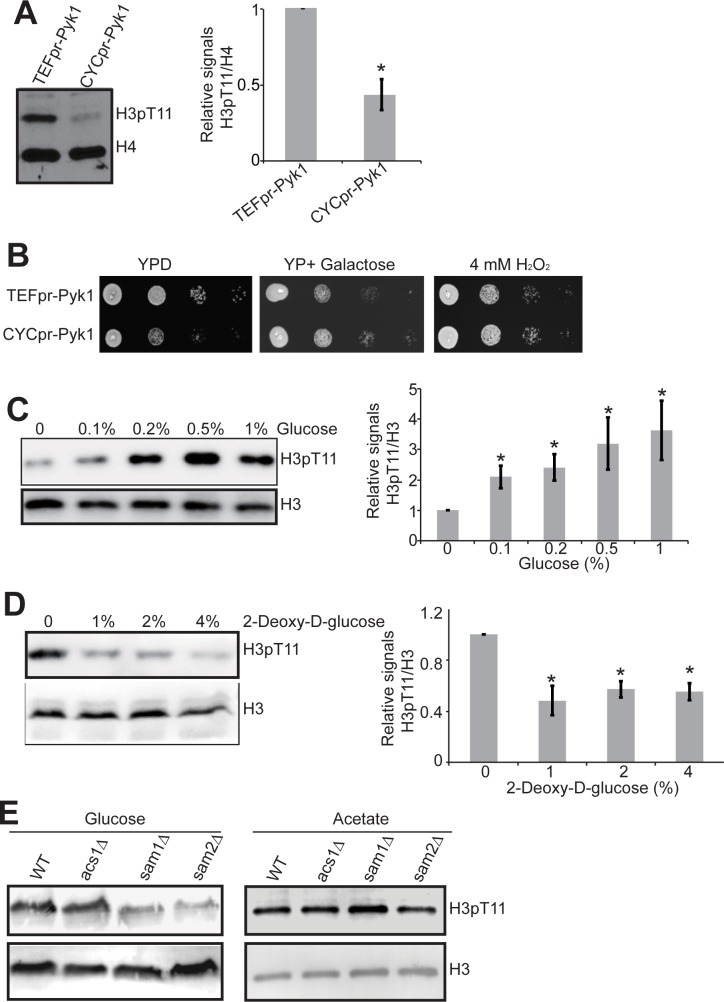
Glucose metabolism regulates H3T11 phosphorylation. (A) Effect of Pyk1 on H3T11 phosphorylation. Left panel: *TEFpr-PYK1* and *CYCpr-PYK1* cells were cultured in YPD medium until OD_600_ of 0.7. Cells were harvested and extracted histones were analyzed by western blots with indicated antibodies. Right panel: Quantitation of western blots in left panel. Shown is the relative intensities of H3pT11/H4 quantified with standard error (SE) (n = 3). *, P<0.05. (B) Lower *PYK1* expression confers oxidative stress resistance. Serial diluted *TEFpr-PYK1* and *CYCpr-PYK1* cells were spotted on YPD, YP+2% galactose or YPD+4mM H_2_O_2_. Shown is the typical example of three independent experiments. (C) Glucose is required for H3T11 phosphorylation. Left panel: Cells were cultured in YP medium, and 0, 0.1%, 0.2%, 0.5% or 1% glucose were then supplied to the medium 3 hours before harvest. Right panel: Quantitation of western blots in left panel. Shown is the relative intensities of H3pT11/H3 quantified with standard error (SE) (n = 3). *, P<0.05. (D) Inhibition of glycolysis by 2-Deoxy-D-glucose reduced H3T11 phosphorylation. Cells were grown in YPD medium with addition of 0, 1%, 2% or 4% 2-Deoxy-D-glucose for 3 hours before harvest. Right panel: Quantitation of western blots in left panel. Shown is the relative intensities of H3pT11/H3 quantified with standard error (SE) (n = 3). *, P<0.05. (E) Glucose was required for SESAME to regulate H3pT11. WT, *acs1Δ*, *sam1Δ*, and *sam2Δ* were grown in YP + 2% glucose or YP + 0.1M potassium acetate. Histones were extracted and analyzed by western blots with indicated antibodies. Histone H3 was a loading control.

Previously, we have used a temperature-sensitive (ts) strain defective in Pyk1 (*cdc19-1*) when grown at 37°C to demonstrated that Pyk1 phosphorylates H3T11 in vivo. Here, we employed the strains that display different *PYK1* expression and activity by expressing *PYK1* under the control of either a strong (*TEF1*) or a weak (*CYC1*) constitutive promoter [16] (S1A Fig). We grew these two strains (*TEF1pr-PYK1*, *CYC1pr-PYK1*) in rich media and examined the global levels of H3T11 phosphorylation by western blot analysis. As shown in Fig 1A, H3T11 phosphorylation was significantly reduced in *CYC1pr-PYK1* strain (Fig 1A), which has reduced *PYK1* expression and lower pyruvate kinase activity (S1A Fig) [16], confirming our previous results that Pyk1 phosphorylates H3T11 in vivo [9]. H3T11 phosphorylation has been shown to confer cells the resistance to oxidative stress [9] and the expression of genes involved in oxidative energy metabolism (*CIT1*, *COX1*) was significantly reduced in H3T11A mutant (S2 Fig). We thus examined the effect of Pyk1 in oxidative stress resistance. In the absence of oxidative stress, *TEF1pr-PYK1* strain grew much better than *CYC1pr-PYK1* in glucose-containing medium due to higher pyruvate kinase activity; however, *CYC1pr-PYK1* grew similar to *TEF1pr-PYK1* when cells were grown in the presence of oxidative stress (Fig 1B), implying that lower expression of *PYK1* could confer cells the resistance to oxidative stress, which is consistant with published results [16]. As H3T11 phosphorylation acts as a feedback mechanism to repress *PYK1* expression [9], it is possible that H3T11 phosphorylation combats oxidative stress by suppressing *PYK1* expression.

It is noteworthy that *TEF1pr-PYK1* and *CYC1pr-PYK1* grew differently when glucose or galactose was used as the sole carbon source (Fig 1B). Hence, we examined the impact of glucose on H3T11 phosphorylation. First, we treated cells with different concentrations of glucose and found that glucose significantly stimulated H3T11 phosphorylation (Fig 1C), indicating that glucose is required for H3T11 phosphorylation. To investigate whether glycolysis is required for H3T11 phosphorylation, we treated cells with glucose analog, 2-Deoxy-D-glucose, which inhibited the activity of hexokinase to phosphorylate glucose and hence suppressed glycolysis. As shown in Fig 1D, the global level of H3T11 phosphorylation was significantly inhibited by 2-Deoxy-D-glucose (Fig 1D, P<0.05), indicating that glycolysis is required for carbohydrate-induced H3T11 phosphorylation.

To further confirm that glycolysis is required for SESAME activity, we grew SESAME mutants (*sam1Δ*, *sam2Δ*) with different carbon sources (glucose and potassium acetate) and then examined their effects on global H3T11 phosphorylation. In contrast to glucose as the sole carbon source, the global levels of H3pT11 were comparable between SESAME mutants and its parental wild type strain when potassium acetate was used as the carbon source (Fig 1E). Together, these data indicate that glycolysis is required for SESAME to phosphorylate H3T11.

### Effect of phosphoglucose isomerase and fructose 1, 6-biphosphate (FBP) on H3T11 phosphorylation

**Fig 2:**
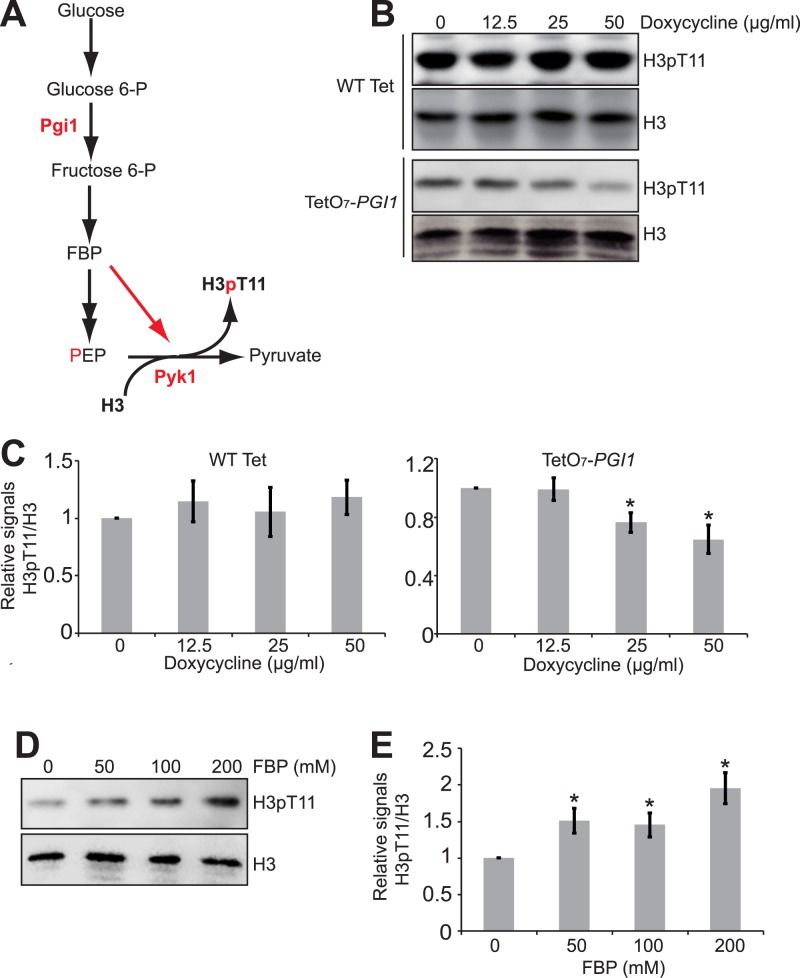
Pgi1 and FBP are required for SESAME-catalyzed H3T11 phosphorylation. (A) Diagram displaying the functions of Pgi1 in glycolysis. (B) Down-regulated *PGI1* leads to reduced H3pT11. Wild type and TetO_7_-*PGI1* mutant was treated with 0, 12.5, 25, and 50 μg/ml doxycycline for 3 hours. Extracted histones were analyzed by western blots with indicated antibodies. (C) Quantitation of western blots in 2B. Shown is the relative intensities of H3pT11/H3 quantified with standard error (SE) (n = 3). *, P<0.05. (D) FBP addition increased H3T11 phosphorylation. Cells were grown in YPD medium with addition of 0, 50, 100, 200 mM FBP for 3 hours before harvest. Extracted histones were analyzed by western blots with indicated antibodies. (E) Quantitation of western blots in (D). Shown is the relative intensities of H3pT11/H3 quantified with standard error (SE) (n = 3). *, P<0.05.

Next, we explored the functions of the glycolysis downstream metabolic enzymes and metabolites in Pyk1-catalyzed H3T11 phosphorylation. Fructose 1, 6-biphosphate (FBP) is an important cofactor for the pyruvate kinase activity of both PKM2 and Pyk1 and depletion of glucose immediately reduces the intracellular level of FBP [17–20]. As an intermediate of glycolysis, FBP has been shown to stimulate the protein kinase activity of PKM2 to phosphorylate H3T11 [21]. To explore the impact of FBP on Pyk1-catalyzed H3T11 phosphorylation, we blocked FBP biosynthesis via down-regulating the expression of *PGI1*, which encodes a phosphoglucose isomerase that catalyzes the conversion of glucose-6-phosphate to fructose-6-phosphate, a precursor for FBP (Fig 2A). We employed the promoter-shutoff strain, TetO_7_-*PGI1*, in which the *PGI1* promoter was replaced with TetO_7_, whose transcription can be shutoff by adding doxycycline [22]. We treated the TetO_7_-*PGI1* mutant with doxycycline to down-regulate *PGI1* transcription from the TetO_7_ promoter (S1B Fig). We also found that down-regulation of *PGI1* expression reduced the intracellular FBP level in TetO_7_-*PGI1* mutant upon doxycycline treatment (S3 Fig, P<0.05). In addition, treatment of TetO_7_-*PGI1* mutant with doxycycline reduced the global H3T11 phosphorylation levels; however, doxycycline has no effect on H3T11 phosphorylation in WT Tet cells (Fig 2B and 2C), indicating that Pgi1 is required for Pyk1-mediated H3T11 phosphorylation.

To directly investigate whether FBP stimulates the protein kinase activity of Pyk1, we treated cells with different amounts of FBP and examined the global levels of H3T11 phosphorylation. FBP significantly stimulated Pyk1-catalyzed H3T11 phosphorylation (Fig 2D and 2E). Thus, like serine and SAICAR (succinyl-5-aminoimidazole-4-carboxamide-1-ribose-5’-phosphate) [9], FBP not only stimulates the pyruvate kinase activity of Pyk1 but also its protein kinase activity to phosphorylate H3T11.

### Effect of enolase and phosphoenonylpyruvate (PEP) on SESAME-mediated H3T11 phosphorylation

**Fig 3:**
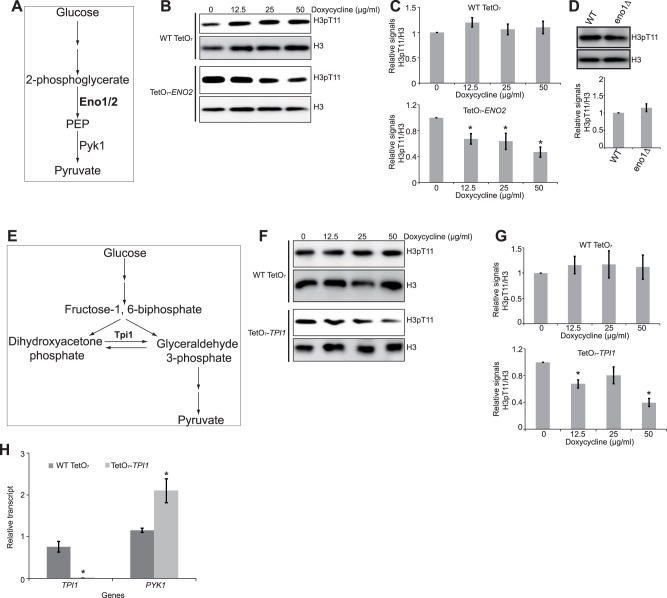
Eno2 and Tpi1 are required for SESAME-mediated H3T11 phosphorylation. (A) Diagram displaying the functions of Eno1/2 in glycolysis. (B) Down-regulated *ENO2* leads to reduced H3pT11. WT Tet and TetO_7_-*ENO2* mutant were treated with 0, 12.5, and 25 μg/ml doxycycline for 3 hours. Histones were extracted and analyzed by western blots with indicated antibodies. Histone H3 was a loading control. (C) Quantitation of western blots in 3B. Shown is the relative intensities of H3pT11/H3 quantified with standard error (SE) (n = 3). *, P<0.05. (D) Deletion of *ENO1* has no effect on H3T11 phosphorylation. Top panel: Analysis of H3T11 phosphorylation in WT and *eno1*∆ by western blots. Extracted histones were analyzed by western blots with indicated antibodies. Bottom panel: Quantitation of western blots in top panel. Shown is the relative intensities of H3pT11/H3 quantified with standard error (SE) (n = 4). P>0.05. (E) Diagram displaying the functions of Tpi1 in glycolysis. (F) Down-regulated *TPI1* leads to reduced H3pT11. WT Tet and TetO_7_-*TPI1* mutant were treated with 0, 12.5, and 25 μg/ml doxycycline for 3 hours. (G) Quantitation of western blots in 3F. Shown is the relative intensities of H3pT11/H3 quantified with standard error (SE) (n = 3). *, P<0.05. (H) *PYK1* transcription was higher in TetO_7_-*TPI1* mutant than wild type. Wild type and TetO_7_-*TPI1* mutant were treated with doxycycline and the expression of *TPI1* and *PYK1* was measured by qRT-PCR. Actin was used as an internal control. Data represent the mean ± S.E. (n = 3). *, P<0.05.

In glycolysis, the presence of glucose promotes the conversion of the PEP to pyruvate by Pyk1 [17–19] (Fig 3A). Since Pyk1 utilized PEP as the donor to phosphorylate H3, we therefore examined the effect of blocking PEP synthesis on H3T11 phosphorylation. We down-regulated the expression of *ENO1*, *ENO2*, which encode enolase converting 2-phosphoglycerate to PEP. The expression of *ENO2* was down-regulated in TetO_7_-*ENO2* mutant upon doxycycline treatment (S1C Fig) [22]. The levels of H3pT11 were reduced upon doxycycline treatment in the TetO_7_-*ENO2* strain but not in WT Tet cells (Fig 3B and 3C). When its paralog, *ENO1* was deleted (S1D Fig), there was no significant change of H3T11 phosphorylation (Fig 3D), implying that Eno2 plays a major role in PEP biosynthesis. As enolase is required for PEP synthesis, it is conceivable that Eno2 regulates H3T11 phosphorylation via PEP.

Gruning et al. showed that reduced Pyk1 activity leads to accumulation of PEP, which in turn inhibits the upper glycolysis enzyme, triosephosphate isomerase (Tpi1) [16]. Tpi1 catalyzes the inter-conversion between glyceraldehyde 3-phosphate and dihydroxyacetone phosphate and its inhibition diverts glycolysis towards pentose phosphate pathway (Fig 3E) [16]. We treated TetO_7_-*TPI1* mutant with doxycycline to reduce *TPI1* transcription from the Tet promoter (S1E Fig). The levels of H3pT11 were reduced upon doxycycline treatment in the TetO_7_-*TPI1* strain but not in WT Tet cells (Fig 3F and 3G), suggesting that Tpi1 is required for H3T11 phosphorylation. As reduced *PYK1* expression confers oxidative stress resistance (Fig 1B), we thus examined the impact of Tpi1 on *PYK1* expression. Our data showed that *PYK1* expression was increased in *TPI1* mutant (Fig 3H), suggesting that Tpi1 could mediate oxidative stress resistance.

### Effect of pyruvate decarboxylase and pyruvate on SESAME-mediated H3T11 phosphorylation

**Fig 4:**
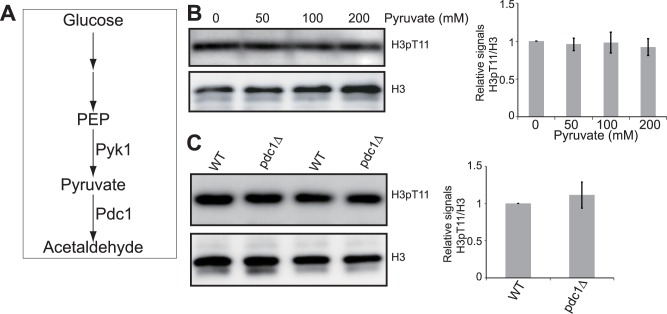
Pyruvate has no effect on SESAME-mediated H3T11 phosphorylation. (A) Diagram displaying the functions of Pdc1 in glycolysis. (B) Addition of pyruvate did not significantly reduce H3T11 phosphorylation. Left panel: Cells were grown in YPD medium with addition of 0, 50, 100, 200 mM pyruvate for 3 hours before harvest. Histones were extracted and analyzed by western blots with indicated antibodies. Histone H3 was a loading control. Right panel: Quantitation of western blots in left panel. Shown is the relative intensities of H3pT11/H3 quantified with standard error (SE) (n = 3). (C) Deletion of *PDC1* did not affect H3T11 phosphorylation. Left panel: Analysis of H3T11 phosphorylation in WT and *pdc1*∆ mutant by western blots. Right panel: Quantitation of western blots in left panel. Shown is the relative intensities of H3pT11/H3 quantified with standard error (SE) (n = 3).

Pyruvate kinase catalyzes the last step of glycolysis, which is also the rate-limiting and irreversible step. We next investigated whether pyruvate as the product of pyruvate kinase (Fig 4A), can feedback inhibit the protein kinase activity to phosphorylate H3T11. We treated cells with different amounts of pyruvate and examined the global levels of H3T11 phosphorylation. As shown in Fig 4B, pyruvate has no inhibitory effect on H3T11 phosphorylation (Fig 4B), consistent with the result that pyruvate kinase catalyzes the irreversible reaction.

We also examined the impact of in vivo accumulation of pyruvate by deleting genes encoding pyruvate decarboxylase, which encodes pyruvate decarboxylase that converts pyruvate to acetaldehyde. H3T11 phosphorylation was not significantly affected in either *pdc1*∆ mutant (Fig 4C) or in *pdc5*∆ mutant (data not shown). Together, these data indicate that pyruvate decarboxylase and pyruvate do not regulate the activity of SESAME to phosphorylate H3T11.

### Effect of folate biosynthesis pathway on SESAME-mediated H3T11 phosphorylation

**Fig 5:**
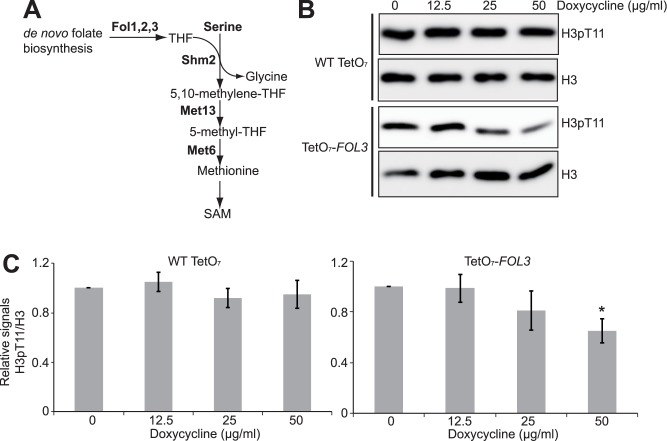
Folate metabolism is required for H3T11 phosphorylation. (A) Diagram displaying *de novo* folate biosynthesis and methionine, SAM biosynthesis. (B) Down-regulated *FOL3* leads to reduced H3pT11. TetO_7_-*FOL3* mutant was treated with 0, 12.5, 25, and 50 μg/ml doxycycline for 3 hours. Extracted histones were analyzed by western blots with indicated antibodies. (C) Quantitation of western blots in 5B. Shown is the relative intensities of H3pT11/H3 quantified with standard error (SE) (n = 3). *, P<0.05.

We have previously reported that histone methyltransferase Set1 stimulates SESAME-catalyzed H3T11 phosphorylation in a SAM-dependent manner and SAM increased global H3K4me3 and H3T11 phosphorylation in a dose dependent manner [9]. Glycolysis-derived serine provides an important methyl source for methionine and SAM synthesis. We have reported that blocking methionine biosynthesis by deletion of *MET6* and *MET13* specifically reduced both H3K4me3 and H3pT11 [9] (Fig 5A). In addition to serine, another critical source for methionine and SAM synthesis is folate and its derivatives tetrahydrofolate (THF) (Fig 5A). Sadhu et al. reported that preventing folate biosynthesis by deleting *FOL3* specifically reduced global H3K4me3 [23]. Since H3K4me3 is directly related to H3T11 phosphorylation, we thus examined the effect of folate metabolism on H3T11 phosphorylation. We used a TetO_7_-*FOL3* mutant and treated it with doxycycline to down-regulate *FOL3* transcription from the Tet promoter (S1F Fig). The levels of H3pT11 were reduced upon doxycycline treatment in the TetO_7_-*FOL3* strain (Fig 5B and 5C), indicating that folate biosynthesis pathway is required for H3T11 phosphorylation.

## Discussion

**Fig 6:**
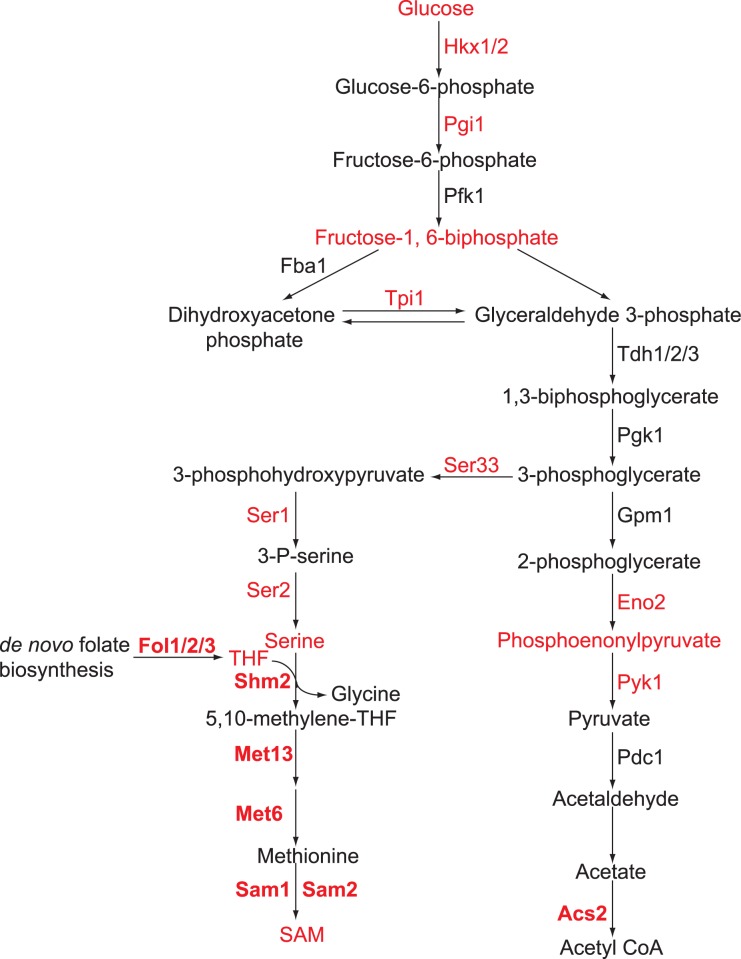
Diagram showing regulation of H3T11 phosphorylation by glycolysis. Metabolic enzymes and metabolites highlighted in red color are required for H3T11 phosphorylation.

Glycolysis is required for global histone acetylation and mono-ubiquitination of H2B at K123 [13,24]. Our work showed that glucose and its metabolism regulates histone H3T11 phosphorylation (Fig 6). Combined with our previous study, we have shown that metabolic enzymes (Hxk1/2, Ser1, Ser2, Ser33, Sam1, Sam2, Pgi1, Tpi1, Eno2, Fol3) and metabolites (FBP, PEP, SAM) regulate Pyk1-mediated H3T11 phosphorylation, providing an intricate connection among glycolysis, histone modification and probably gene expression. Since glycolytic enzymes and metabolites are highly conserved from yeast to mammalian cells, it is conceivable that glycolysis regulates PKM2-mediated H3T11 phosphorylation via its metabolic enzymes and generated metabolites. Hence, our study provides insights into the connection between glycolysis and histone modifications and most importantly, provides one plausible explanation of the “Warburg effect”.

Gruning et al. have shown that lower Pyk1 activity increases antioxidative capacity [16] and our data confirmed this conclusion (Fig 1B). We have previously reported that H3T11 phosphorylation inhibits *PYK1* expression and confers cells the resistance to oxidative stress [9]. Based on this information, we proposed that nucleus Pyk1-catalyzed H3T11 phosphorylation represses *PYK1* expression, which in turn stimulates flux towards pentose phosphate pathway. As a consequence, redox metabolism is enhanced to prevent the accumulation of reactive oxygen species (ROS) (S4 Fig). Nevertheless, much work has to be done to prove this model.

Glucose metabolism is required for SESAME-mediated H3T11 phosphorylation. Depletion of glucose or inhibition of glycolysis by 2-Deoxy-D-glucose significantly reduced H3T11 phosphorylation (Fig 1C and 1D). When cells were grown with acetate as the carbon source, H3T11 phosphorylation was not significantly altered in SESAME mutants, implying that H3T11 phosphorylation was not regulated by SESAME under these circumstances. SESAME required glucose-derived metabolites to catalyze enzymatic reactions.

One contribution of glycolysis to H3T11 phosphorylation is providing the substrate, PEP for Pyk1. Down-regulating the expression of *ENO2* reduced the global levels of H3T11 phosphorylation (Fig 3C). Although there are two genes encoding enolase (*ENO1*, *ENO2*), only *ENO2* is required for H3T11 phosphorylation. The role of Eno2 in regulating H3T11 phosphorylation is probably related to their abundance with 20-fold higher Eno2 than Eno1 when glucose is used as the carbon source [25]. However, adding PEP into YPD rich media failed to stimulate Pyk1-mediated H3T11 phosphorylation (data not shown). This is probably caused by the dissociation constant (*Km*) of Pyk1 to PEP is very low, approximately 0.3 mM [20].

Another contribution of glycolysis to H3T11 phosphorylation is supplying the cofactor FBP for Pyk1. It is well-known that FBP stimulates Pyk1 to convert PEP to pyruvate [20]. FBP has been shown to activate both the pyruvate kinase and protein kinase activity of PKM2 [21]. Here, we found that FBP also activates the protein kinase activity of Pyk1 to phosphorylate H3T11 (Fig 2E). Hence, the stimulatory effect of FBP on pyruvate kinase-catalyzed H3T11 phosphorylation is quite conserved from yeast to tumor cells.

Together, we identified three major contribution of glucose metabolism to H3T11 phosphorylation: 1. Glycolysis provides the substrate PEP; 2. Glycolysis supplies cofactor FBP; 3. Glycolysis promotes the *de novo* synthesis of serine. On one hand, serine derived from glycolysis contributes to H3T11 phosphorylation by acting as a coactivator for Pyk1; on the other hand, serine can be fueled to SAM synthesis and facilitate H3K4me3, which then enhanced the ability of Pyk1 to phosphorylate H3T11 via a cross-talk between Set1 and SESAME [9]. Given the fact that yeast and cancer cells prefer aerobic glycolysis and PKM2 and H3T11 phosphorylation play important role in regulating “Warburg effect” and tumor progression [26,27], understanding how glycolysis modulates SESAME activity is important in understanding the Warburg effect and the connection between glycolysis and gene expression.

## Supporting information

S1 Fig Confirmation of gene mutants used in this study. (A) *PYK1* was expressed at a lower level in *TEFpr-PYK1* than *CYC1pr-PYK1*. Actin was used as an internal control. Data represent the mean ± SE (n = 3). *, P<0.05. (B) The expression of *PGI1* was significantly down-regulated in TetO7-*PGI1* mutant by doxycycline. (C) The expression of *ENO2* was significantly down-regulated in TetO7-*PGI1* mutant by doxycycline. (D) *ENO1* was deleted in *eno1*∆ mutant. The genome of WT and *eno1*∆ mutant were extracted. The deletion of *ENO1* was confirmed by PCR using *ENO1* specific primers. *ACT1* primers were used as an internal control. (E) The expression of *PGI1* was significantly down-regulated in TetO7-*PGI1* mutant by doxycycline. (F) The expression of *PGI1* was significantly down-regulated in TetO7-*PGI1* mutant by doxycycline. All experiments except S1D Fig were measured by qRT-PCR. Data represent the mean ± SE of three independent experiments. *, P<0.05; **, P<0.01; ***, P<0.001.
(EPS)

S2 Fig The expression of *CIT1* and *COX1* was reduced in H3T11A compared with wild type H3 (H3). Actin was used as an internal control. Data represent the mean ± SE (n = 3). *, P<0.05.
(EPS)

S3 Fig Down-regulation of *PGI1* reduced intracellular FBP concentrations. (A) The expression of *PGI1* was significantly down-regulated in TetO7-*PGI1* mutant by doxycycline treatment. (B) Down-regulated *PGI1* leads to reduced intracellular FBP concentrations. Data represent the mean ± SE (n = 3). *, P<0.05; ***, P<0.001.
(EPS)

S4 Fig Proposed roles of Pyk1-catalyzed H3T11 phosphorylation in oxidative stress resistance. (A) Around 1.9% Pyk1 is localized in nucleus and this nucleus Pyk1 catalyzed H3T11 phosphorylation [9]. H3T11 phosphorylation in turn repressed *PYK1* expression. Reduced *PYK1* confers cells resistance to oxidative stress by stimulating pentose phosphate pathway, which increased antioxidative metabolism and prevents ROS accumulation. (B) Model explains oxidative stress resistance in *CYC1pr-PYK1* mutant. In *CYC1pr-PYK1* mutant, the protein level of Pyk1 was low, which increased flux towards pentose phosphate pathway to gain resistance to oxidative stress. (C) Model explains oxidative stress resistance in H3T11A mutant. In H3T11A mutant, *PYK1* expression was up-regulated, which attenuates flux towards pentose phosphate pathway and reduces resistance to oxidative stress.
(EPS)

S1 Table Primers used in this study. (DOC)
