# Supplementary material for: Regulation of SESAME-mediated H3T11 phosphorylation by glycolytic enzymes and metabolites
Source: PLoS One. 2017 Apr 20;12(4):e0175576. doi: 10.1371/journal.pone.0175576 (PMC5398556; doi:10.1371/journal.pone.0175576)
Supplement: S1 Table — (DOC) [file pone.0175576.s005.doc]

**Table S1 List of primers used in this study**

| **Gene name** | **Sequence** |
| --- | --- |
| *ACT1* | CTGTCGAGAGATTTCTCTTTTACC |
|  | GCCCCTATTTATTCCAATAATATCG |
| *PYK1* | GTCCGTATGAACTTCTCTCACG |
|  | GTGGGATTGGGTAGTCAACATC |
| *ENO1* | GGCCGTCGATGACTTCTTGA |
|  | CAGCGAAGGTCTTAGCACCA |
| *ENO2* | CTCCAAACATTCAAACCGCTG |
|  | GTCGTACTTACCGTCCTTGAAG |
| *TPI1* | AGAAGCCACAAGTCACTGTC |
|  | ACCCACTTAGCACCAACATC |
| *PGI1* | TCTGGTCGGCTATTGGTTTG |
|  | TTGGGTGAAGTGGTTGTCG |
